# Morphine activation of mu opioid receptors causes disinhibition of neurons in the ventral tegmental area mediated by β-arrestin2 and c-Src

**DOI:** 10.1038/s41598-017-10360-8

**Published:** 2017-08-30

**Authors:** Fiona A. Bull, Daniel T. Baptista-Hon, Jeremy J. Lambert, Wendy Walwyn, Tim G. Hales

**Affiliations:** 1 The Institute of Academic Anaesthesia, University of Dundee, Dundee, DD1 9SY UK; 2 Division of Neuroscience, School of Medicine, Ninewells Hospital, University of Dundee, Dundee, DD1 9SY UK; 3 Shirley and Stefan Hatos Center for Neuropharmacology, University of California, Los Angeles, California, 90095 USA

## Abstract

The tyrosine kinase, c-Src, participates in mu opioid receptor (MOP) mediated inhibition in sensory neurons in which β-arrestin2 (β-arr2) is implicated in its recruitment. Mice lacking β-arr2 exhibit increased sensitivity to morphine reinforcement; however, whether β-arr2 and/or c-Src participate in the actions of opioids in neurons within the reward pathway is unknown. It is also unclear whether morphine acts exclusively through MOPs, or involves delta opioid receptors (DOPs). We examined the involvement of MOPs, DOPs, β-arr2 and c-Src in the inhibition by morphine of GABAergic inhibitory postsynaptic currents (IPSCs) recorded from neurons in the mouse ventral tegmental area. Morphine inhibited spontaneous IPSC frequency, mainly through MOPs, with only a negligible effect remaining in MOP−/− neurons. However, a reduction in the inhibition by morphine for DOP−/− *c.f*. WT neurons and a DPDPE-induced decrease of IPSC frequency revealed a role for DOPs. The application of the c-Src inhibitor, PP2, to WT neurons also reduced inhibition by morphine, while the inactive PP3, and the MEK inhibitor, SL327, had no effect. Inhibition of IPSC frequency by morphine was also reduced in β-arr2−/− neurons in which PP2 caused no further reduction. These data suggest that inhibition of IPSCs by morphine involves a β-arr2/c-Src mediated mechanism.

## Introduction

The ventral tegmental area (VTA) is important for the development of reward and goal directed behaviours^1^. Mu opioid receptors (MOPs) are required for the reinforcing properties of morphine^2^. The activation of MOPs on presynaptic GABAergic neurons in the VTA reduces the frequency of inhibitory postsynaptic currents (IPSCs) in dopaminergic neurons, an effect thought to contribute to increased dopamine release in the striatum^3–8^. Activated MOPs couple to several effectors that might mediate inhibition of IPSCs, however, the mechanisms involved in the VTA have not been elucidated.

MOP activation liberates G protein αi/o and βγ subunits, which modulate several effectors, including voltage-activated Ca^2+^ channels (VACCs), G-protein activated inwardly rectifying K^+^ (GIRK) channels and adenylyl cyclase^8^. In addition, MOP activation also recruits arrestins 2 and 3 (β-arrestin 1 and 2), which participate in G protein couple receptor (GPCR) desensitisation, endocytosis and signalling through various kinases such as ERK1/2, Akt, JNK and c-Src^9, 10^. Opioid induced stimulation of the canonical G protein signalling system in GABAergic neurons is thought to be responsible for disinhibition of VTA dopaminergic neurons. Opioids activate GIRK channels in presynaptic GABAergic neurons; however, neither the pharmacological blockade of these channels, nor the abolition of action potentials prevents disinhibition of VTA dopaminergic neurons^7^. This finding implicates presynaptic VACCs and/or other components of vesicular trafficking as targets for MOP-mediated inhibition of presynaptic GABA release. N-type VACCs are likely candidates as their selective blockade in the VTA disinhibits dopaminergic neurons leading to increased accumbal dopamine release^11^.

The mechanism of opioid inhibition of neurotransmitter release is relatively well established for sensory neurons in which reduced Ca^2+^ entry through VACCs is a key component^12^. MOP activation liberates G protein βγ subunits, which bind directly to VACCs leading to inhibition^13^. MOP activation also inhibits N-type VACCs through a βγ-independent pathway mediated by the non-receptor tyrosine kinase, c-Src^14^, which is highly enriched in neuronal terminals^15^. MOP agonists activate c-Src through β-arrestin dependent and independent mechanisms^16, 17^. In dorsal root ganglion (DRG) neurons activation of c-Src by the MOP agonist DAMGO requires β-arrestin2^18^. Mice lacking β-arrestin2 (β-arr2−/−) exhibit reduced morphine analgesic tolerance and persistent MOP-mediated analgesia *via* constitutive MOP activity^19, 20^.

While MOPs are required for the reinforcing and analgesic actions of morphine, delta opioid receptors (DOPs) also have a role in these behaviours^2, 21–23^. The existence of MOPs in the VTA is well established, however, there is less evidence regarding DOPs. The recent use of red-MOP/green-DOP, double knock-in mice identified expression of both receptors in several brain nuclei including the VTA^24^. The observation that rats self-administer the DOP agonist, DPDPE, into the VTA suggests that these DOPs are functional^25^. However, the contribution of DOPs to morphine-mediated behaviors is unclear. DOP−/− mice exhibit impaired morphine CPP^22, 23^. However, this deficit appears to be one of morphine-context association, rather than a loss of reinforcement and therefore a role for DOPs in this behavior is uncertain.

Given the importance of morphine-induced disinhibition in the VTA in influencing accumbal dopamine release and subsequent reinforcement, we have used whole-cell voltage-clamp to investigate opioid modulation of GABA-mediated IPSCs in VTA neurons. Using genetically modified mice and pharmacological intervention we examined the involvement of MOPs, DOPs, β-arrestin2 and c-Src to better understand opioid-mediated signalling in this key location within the reward pathway. Importantly, our data confirm the involvement of MOPs, identify a role for DOPs and reveal that the β-arrestin2/c-Src signalling pathway mediates a component of the inhibitory action of morphine on GABAergic neurotransmission.

## Results

### MOP-mediated inhibition of spontaneous IPSCs in mouse VTA neurons

More than two thirds of neurons in the mouse VTA are dopaminergic, with approximately one third being GABAergic and 2% glutamatergic^1^. While no morphology is unique to these neuronal subtypes, the prevalence of soma shapes differs between GABAergic and dopaminergic neurons; more than 85% of elliptical neurons are tyrosine hydroxylase positive and more than 60% of multipolar neurons are GAD67 positive^26^. While, dopaminergic neurons in the VTA can be identified immunohistochemically, there is less certainty regarding their identification by electrophysiological characteristics^3, 4, 7, 26–31^.

**Figure 1:**
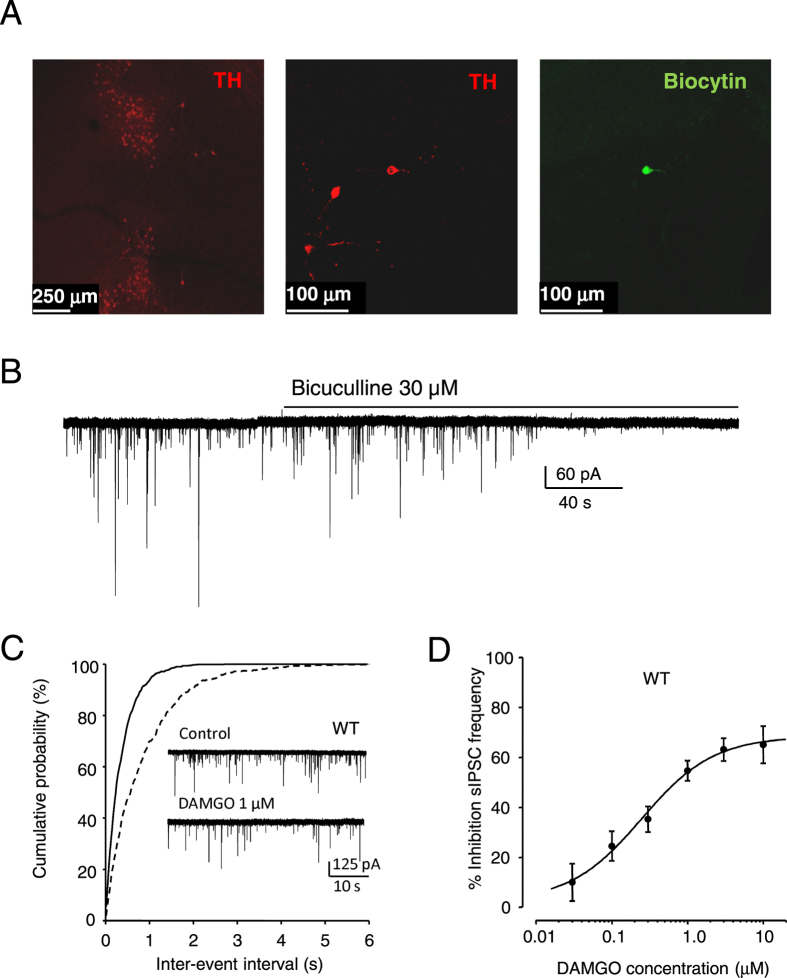
MOP-mediated inhibition of sIPSCs in mouse VTA neurons. (**A**) *Left panel*, Confocal microscopy image of a brain slice labelled with antibody to TH (secondary antibody conjugated to Alex Fluor 594). The pattern of TH staining corresponds to the VTA. *Middle panel*, TH positive dopaminergic cell within the VTA, which was (*right panel*) also positive for Biocytin (streptovidin secondary antibody conjugated to Alexa Fluor 488). (**B**) Bicuculline (30 μM) inhibited sIPSCs indicating that these events are mediated by GABA_A_ receptors. This recording is representative of seven similar experiments. (**C**) Exemplar recording of the inhibition of sIPSC frequency by DAMGO (1 μM) in a VTA neuron, with the associated graph of the cumulative probabilities of inter-event intervals. (**D**) Graph of the concentration-response relationship for inhibition of sIPSC frequency by DAMGO (EC_50_ = 0.25 µM (95% CIs = 0.05 and 1.3 µM), slope = 0.7, n = 4–7). The symbols represent the mean and vertical lines are ± SEM.

Initially, we immunohistochemically labelled horizontal slices containing the VTA with a tyrosine hydroxylase (TH) antibody and a secondary antibody conjugated to Alexa Fluor 594 (Fig. 1A). Elliptical VTA neurons were targeted for whole-cell voltage-clamp recording of IPSCs. When biocytin (1%) was included in the recording electrode subsequent exposure to streptavidin-Alexa Flour 488 secondary antibody revealed fluorescence in cells that were TH positive when imaged using confocal microscopy (Fig. 1A, middle and left panels, respectively). In keeping with a previous report^26^ the majority of TH positive cells, including the biocytin labelled cell in this example, exhibited a characteristic elliptical shape and were not multipolar. Spontaneous IPSCs recorded from such cells exhibited an average baseline frequency, determined from the inter-event interval, of 2.1 ± 0.2 Hz, (n = 69). The application of TTX (500 nM) to the recording solution did not significantly affect sIPSC frequency (Supplementary Figure 1A). Such events were abolished by bicuculline (30 µM), confirming that they are GABAergic (Fig. 1B). There was no change in basal holding current during the application of bicuculline suggesting that there was a lack of tonic GABA_A_-receptor mediated inhibition. Peak amplitudes, rise times, times to decay to 70% of peak amplitude (T_70_) and the decay time constants (τw) were not significantly different for sIPSCs compared to the mIPSCs recorded in the presence of TTX, suggesting that, under these conditions, there is little action potential dependent release ongoing (Supplementary Table 1). Both IPSC frequency and T_70_ for decay are similar to those reported previously in recordings from VTA dopaminergic neurons^32–34^. Furthermore, the T_70_ for decay differed from that observed previously in GABAergic neurons of the VTA^33^. Taken together these data suggest that recordings were predominantly from dopaminergic neurons.

The selective MOP agonist, DAMGO (1 µM) inhibited the frequency of sIPSCs in all cells tested, by on average 55 ± 4% (n = 7) recorded from WT VTA neurons (Fig. 1C,D). The inhibitory effect of DAMGO is similar in magnitude to that observed previously in mouse VTA neurons^28, 34^. The application of DAMGO (0.03–10 µM) caused a concentration-dependent inhibition of sIPSC frequency (Fig. 1D).

### Morphine inhibits IPSCs through activation of MOPs and DOPs

**Figure 2:**
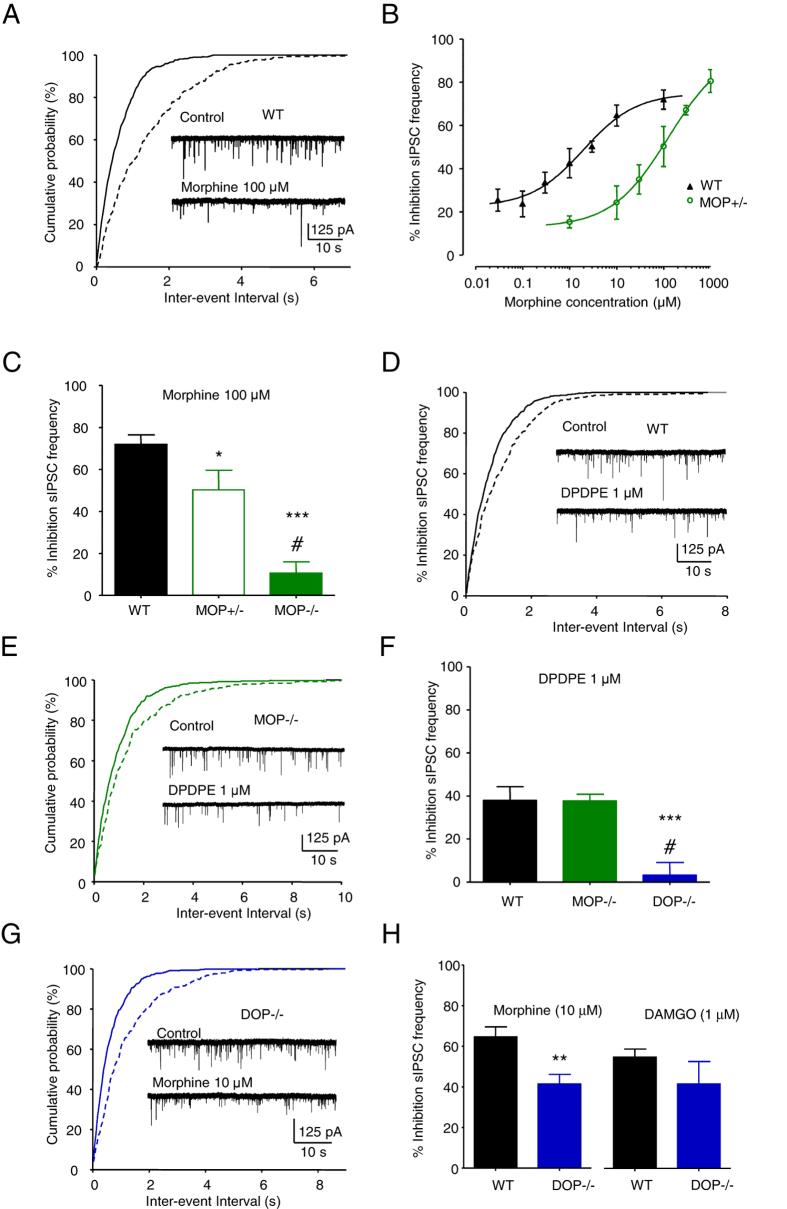
Morphine inhibits IPSCs through activation of MOPs and DOPs. (**A**) Exemplar recording of the inhibition of sIPSC frequency by morphine (100 μM) in a VTA neuron, with the associated graph of cumulative probability of the inter-event intervals. (**B**) Graph of the concentration-dependent inhibition of sIPSC frequency by morphine in WT neurons (EC_50_ = 2 µM (95% CIs = 0.54 and 7.2 µM), slope = 0.8, n = 5–10) and MOP+/− neurons (EC_50_ = 128 µM, (95% CIs = 20 and 826 µM), slope = 0.7, n = 4–8). (**C**) Bar graph of the average inhibition by morphine (100 μM) of sIPSC frequency in WT (72 ± 5%, n = 7), MOP+/− (50 ± 9%, n = 4) and MOP−/− (11 ± 5%, n = 7) neurons. (**D**) Exemplar sIPSCs recorded from a WT neuron in the absence and presence of DPDPE (1 µM), with the associated graph of cumulative probabilities. (**E**) Exemplar recording from a MOP−/− neuron in the absence and presence of DPDPE, with the graph of cumulative probability. (**F**) Bar graph illustrating the average inhibition by DPDPE of sIPSC frequency in WT (37 ± 7%, n = 6), MOP−/− (34 ± 2%. n = 5) and DOP−/− neurons (7 ± 5%, n = 6). (**G**) sIPSCs recorded from a DOP−/− VTA neuron, illustrating the remaining inhibition by morphine, with the associated graph of cumulative probability. (**H**) Bar graph of inhibition of sIPSC frequency by morphine and DAMGO. Inhibition by morphine was reduced in DOP−/− neurons (42 ± 5%, n = 8) compared to WT neurons (64 ± 5, n = 10; unpaired t test p = 0.004; see Table 1). While Inhibition by DAMGO (42 ± 11%, n = 8) was not significantly different (unpaired t test p = 0.31, WT n = 7, DOP−/− n = 7) to WT (55 ± 4%, n = 7). Vertical lines represent ± SEM. One way ANOVA p < 0.001, *post hoc* Tukey test results are shown on the graphs. *p < 0.05, **p < 0.01, ***p < 0.001.

Morphine also inhibited sIPSC frequency when applied to WT VTA neurons (Fig. 2A). This inhibition was unaffected by the presence of TTX (500 nM) and occurred without alteration of the amplitude, or kinetics of IPSCs (Supplementary Figure 1 and Supplementary Table 1). The inhibition of sIPSC frequency by morphine and DAMGO did not exhibit reversal after 300 s of wash (data not shown). However, partial reversal of inhibition by morphine (10 µM) was observed within 300 s of application of the MOP antagonist naloxone (10 µM) in recordings of sIPSCs from 4 of 6 neurons tested (Supplementary Figure 2).

Consistent with a role for MOPs in the inhibition of sIPSC frequency by morphine, its application to slices from MOP+/− mice caused a significantly less potent inhibition of sIPSC frequency (Fig. 2B; Table 1). Furthermore, inhibition of sIPSC frequency by morphine (100 µM) was greatly reduced in MOP−/− neurons (Fig. 2C). While electrophysiological recording from MOP+/− and MOP−/− VTA neurons revealed altered responses to morphine, there was no difference between the basal sIPSC frequency compared to recordings from WT neurons (Supplementary Figure 3A).

**Table 1:** Frequency of sIPSCs recorded from VTA neurons in WT and knockout mice.

Genotype	Control sIPSC frequency (Hz)	Morphine sIPSC frequency (Hz)	% Inhibition
WT	2.9 ± 0.5	1.0 ± 0.3	64 ± 5 (n = 10)
MOP−/−	1.5 ± 0.4	1.2 ± 0.3	14 ± 5 (n = 5)*
DOP−/−	1.6 ± 0.4	0.9 ± 0.3	44 ± 4 (n = 7)^†^
BAR2−/−	2.2 ± 0.3	1.7 ± 0.2	23 ± 4 (n = 5)*
BAR2−/−//DOP−/−	2.1 ± 0.4	1.5 ± 0.3	24 ± 9 (n = 6)*

Morphine inhibits sIPSC frequency, but does not alter the kinetics of the events (see Supplementary Table 1). While there was no effect of genotype on control sIPSC frequency (one way ANOVA, also see Supplementary Figure 1C for combined data of all genotypes), the frequency of events were significantly decreased following morphine exposure compared to control (paired Student’s t test, p < 0.05) for all genotypes except MOP−/− (p = 0.08). In recordings from MOP−/− VTA neurons, 100 µM morphine was applied, in all other cases 10 µM morphine was used. One way ANOVA with *post hoc* Tukey’s test: ^†^DOP−/− vs MOP−/−, p < 0.05; *Different from WT, p < 0.005.

The selective DOP agonist, DPDPE (1 µM), inhibited sIPSC frequency recorded from WT VTA neurons (Fig. 2D). An absence of MOPs had no effect on the inhibition by DPDPE, which inhibited sIPSC frequency recorded from MOP−/− neurons to a similar extent compared to WT neurons (Fig. 2E,F). By contrast, DPDPE (1 μM) had no significant effect on sIPSC frequency when applied to DOP−/− neurons (Fig. 2F). These observations confirm the presence of DOPs within the mouse VTA, which can function independently of MOPs.

The existence of DOPs in the VTA raises the possibility that they may contribute to the inhibitory effect of morphine on IPSC frequency. Morphine (10 μM) inhibited the sIPSC frequency recorded from DOP−/− VTA neurons (Fig. 2G). However, the inhibition by morphine in DOP−/− neurons was significantly reduced when compared to WT neurons (Fig. 2H). By contrast, the inhibition of sIPSC frequency by DAMGO was not significantly different in the WT and DOP−/− VTA neurons consistent with the agonist being selective for MOPs (Fig. 2H).

While electrophysiological recording from DOP−/− VTA neurons revealed altered responses to DPDPE and morphine, there was no difference between the basal sIPSC frequency compared to recordings from WT neurons (Supplementary Figure 3A).

### A role for c-Src in opioid inhibition of sIPSC frequency

**Figure 3:**
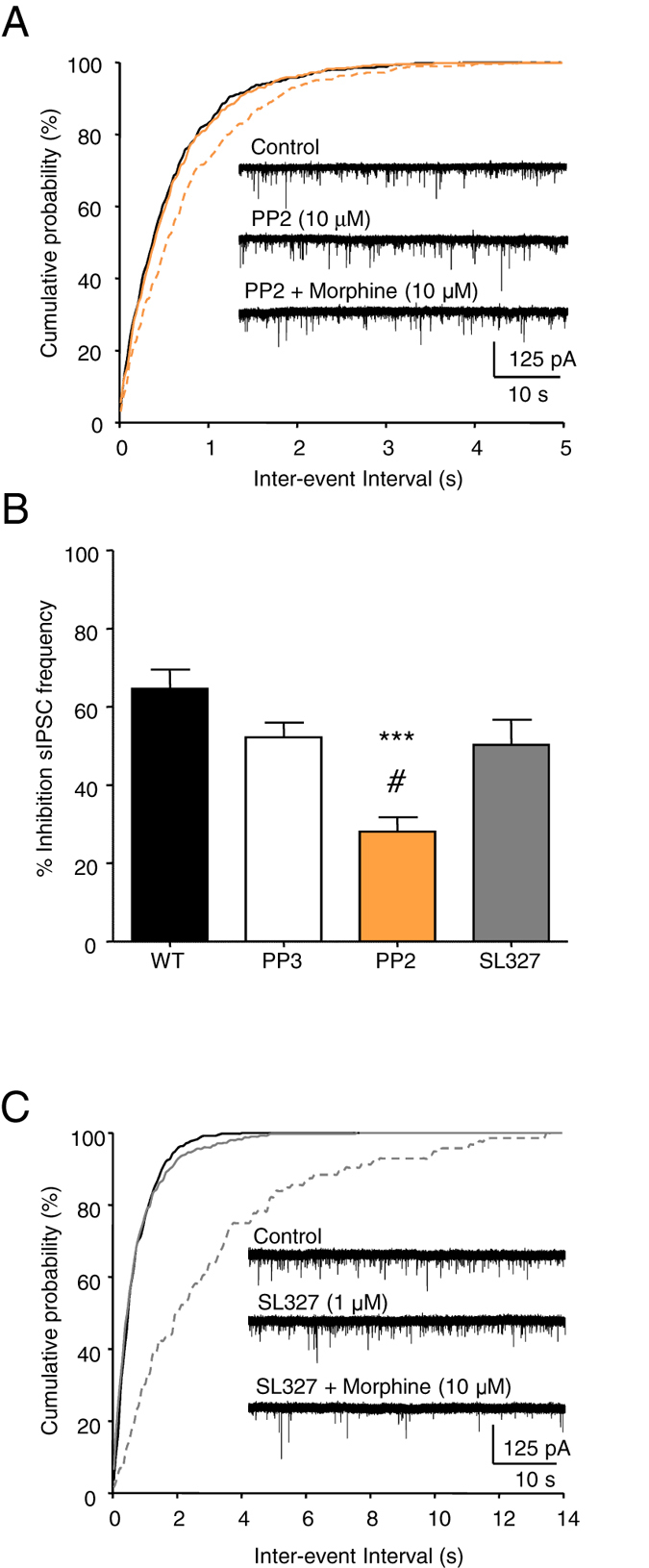
Inhibition of c-Src attenuates the reduction in sIPSC frequency by morphine. (**A**) Exemplar sIPSCs recorded in the absence of drugs and in the presence of PP2 alone or PP2 plus morphine, with the associated graph of the cumulative probabilities of inter-event intervals. (**B**) The bar graph reveals that PP2 reduced the inhibition of sIPSC frequency by morphine (to 28 ± 4%, n = 6), while the inactive analogue, PP3 (n = 6), and the MEK inhibitor, SL327 (n = 7), had no effect (52 ± 4% and 50 ± 6%, respectively). (**C**) Exemplar currents recorded in the absence of any drug and in the presence of SL327 alone or SL327 plus morphine with the associated graph of the cumulative probabilities. PP2, PP3 and SL327 had no effect on sIPSC frequency when applied alone (see Supplementary Figure 4D–F). One way ANOVA, *post hoc* Tukey test, ***p < 0.001 (PP2 vs WT), ^#^p < 0.05 (PP2 vs PP3 and SL327).

Previous studies have identified a role for c-Src in the coupling of MOPs to VACCs in DRG neurons^14, 18^. We investigated whether c-Src participates in the inhibitory effect of morphine on sIPSC frequency in VTA neurons, using the c-Src inhibitor PP2 and its inactive analogue PP3^18^. Neither the application of PP2 (10 μM; Fig. 3A), nor PP3 (10 μM) affected sIPSC frequency recorded from WT VTA neurons (Supplementary Figure 3B,C). However, when PP2 (10 μM) was applied with morphine (10 μM; Fig. 3A) there was a reduction in the inhibition of sIPSC frequency in VTA neurons compared to those that received morphine alone, or morphine in the presence of PP3 (Fig. 3B). By contrast, the inhibition of sIPSC frequency by morphine was not significantly different in the presence or absence of PP3 (Fig. 3B).

ERK activation has been implicated in the psychomotor stimulatory effects of morphine in mice^35^. Systemic administration of the MEK inhibitor, SL327, to mice inhibits the morphine-evoked locomotor response. SL327 inhibits activation of ERK in the mouse striatum^36^. We investigated whether the MEK-ERK pathway is involved in the inhibition of sIPSC frequency by morphine in the VTA. SL327 (1 μM), had no effect on sIPSC frequency when applied alone (Fig. 3C; Supplementary Figure 3D). Furthermore, the inhibition of sIPSC frequency by morphine (10 μM) applied in the presence of SL327 (1 μM) was not significantly different from control (Fig. 3B).

### A role for β-arrestin2 in opioid inhibition of sIPSC frequency

**Figure 4:**
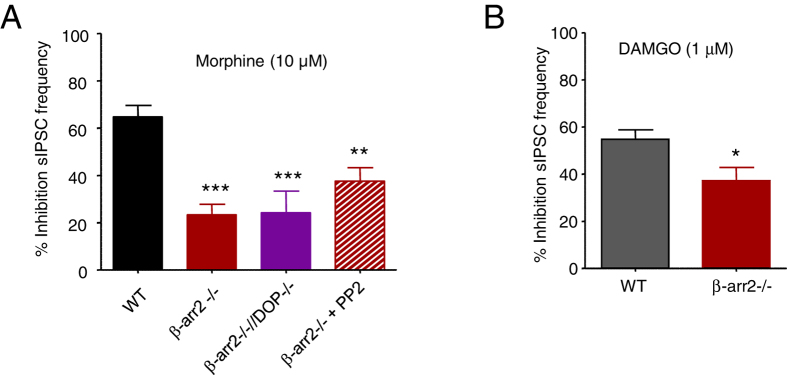
The absence of β-arrestin2 reduces inhibition of sIPSC frequency by opioids. (**A**) Bar graph illustrating the effect of reduced β-arrestin2 on the inhibition of sIPSC frequency by morphine (10 μM) in the VTA. Morphine (10 μM) caused inhibitions of sIPSC frequency by 65 ± 5% (n = 10) and 23 ± 4% (n = 5) in WT and β-arr2−/− neurons, respectively. Neither the additional knock out of DOPs (β-arr2−/−//DOP−/−), nor the application of the c-Src inhibitor PP2 (10 µM), n = 4 and 5, respectively, caused any additional inhibition of the effect of morphine (24 ± 9%, n = 6 and 38 ± 6%, n = 6, respectively). These data are consistent with the idea that c-Src and DOP dependent components of morphine’s inhibition require β-arrestin2. One way ANOVA, *post hoc* Dunnett’s test, **p < 0.01 and ***p < 0.001. (**B**) The inhibition of sIPSC frequency by DAMGO (1 µM) was also significantly reduced in β-arr2−/− VTA neurons (37 ± 6%, n = 8) compared to WT neurons (55 ± 4% n = 7; unpaired t test, *p < 0.05).

The MOP-mediated activation of c-Src in DRG neurons requires β-arrestin2^18^. We examined the effect of morphine (10 μM) on sIPSC frequency in β-arr2−/− VTA neurons (Fig. 4A). A reduction in the inhibitory effect of morphine was observed in β-arr2−/− mice (Fig. 4A; Table 1).

When co-expressed, recombinant MOP and DOP colocalise in the cell surface membrane with β-arrestin2 in the absence of agonist, suggesting the occurrence of constitutive recruitment of β-arrestin2^37^. We investigated whether the simultaneous expression of MOP and DOP affects β-arrestin2 recruitment using the PathHunter protein complementation assay (Supplementary Figure 4). DAMGO and morphine both stimulated the recruitment of β-arrestin2 to MOPs, with DAMGO being the more potent and efficacious of the two agonists (Supplementary Figure 4A). The inclusion of DOPs reduced the potency of DAMGO and morphine (Supplementary Figure 4A) and enhanced the agonist-independent recruitment of β-arrestin2 to MOPs (Supplementary Figure 4B). This demonstrates DOPs enable constitutive β-arrestin2 recruitment to MOPs. A lack of such β-arrestin2 recruitment could be responsible for the deleterious effect on the morphine-evoked inhibition of sIPSC frequency observed in DOP−/− VTA neurons (Fig. 2F). Alternatively, the deletion of β-arrestin2 and DOP might hinder the inhibitory action of morphine through independent mechanisms.

We bred β-arr2−/− mice with DOP−/− mice to generate β-arr2−/−//DOP−/− double knockout mice. These mice had no overt phenotype that distinguished them from WT mice, or from either of the individual founder knockout strains. Morphine (10 μM) caused a 24 ± 9% (n = 6) reduction in sIPSC frequency when applied to β-arr2−/−//DOP−/− VTA neurons (Fig. 4A). The apparent lack of additivity of the deleterious effects of an absence of DOPs and β-arrestin2, on the inhibition by morphine, implies that the roles of these two proteins in this regard are not independent (Table 1). This is consistent with a role for DOPs in morphine-evoked β-arrestin2-mediated signalling in VTA neurons. However, the inhibitory effect of DAMGO on sIPSC frequency was also slightly diminished in β-arr2−/− VTA neurons (Fig. 4B), despite a lack of involvement of DOPs in this effect (Fig. 2D). This suggests that MOPs can signal *via* β-arrestin2 in VTA neurons independently of DOPs.

We also applied PP2 to β-arr2−/− VTA neurons to determine whether the inhibitory effects of a lack of β-arrestin2 and the c-Src inhibitor were additive (Fig. 4A). The deleterious effect of the combination of c-Src inhibition and a lack of β-arrestin2 on morphine’s inhibition of sIPSC frequency was no different to that of a lack of β-arrestin2 alone.

## Discussion

This study demonstrates that opioid receptor activation in VTA neurons causes inhibition of GABAergic sIPSC frequency *via* a β-arrestin2 and c-Src mediated mechanism. The inhibition by morphine was reduced to a negligible level in MOP−/− VTA neurons implying that the majority of the response is mediated by MOPs. Furthermore, consistent with receptor reserve in the VTA^38^, the absence of the full complement of MOPs (in MOP+/− neurons) significantly reduced the potency of morphine without affecting efficacy. There is also a more minor role for DOPs in the inhibition of sIPSC frequency by morphine as evidenced by its reduction in DOP−/− VTA neurons. The existence of DOPs in the VTA was also supported by an inhibitory action of DPDPE on sIPSC frequency in WT and MOP−/− slices that was absent from DOP−/− slices.

By contrast to morphine evoked inhibition of sIPSC frequency, which was reduced in MOP−/−, DOP−/−, β-arr2−/− and PP2 treated WT neurons, basal sIPSC frequency was unaffected by these manipulations compared to control neurons. We are unaware of prior studies of DOP−/− or β-arr2−/− VTA neurons. However, there is a previous report of MOP−/− VTA neurons in which sIPSC frequency was elevated compared to WT neurons^39^. There are several important differences between our approach and that of Mathon and colleagues, including the targeting strategy used for establishing MOP−/− mice and the background strain. Furthermore, mice used for generating VTA slices in the earlier study tended to be younger and recordings were made at room temperature in contrast to our recordings at 36 °C.

We previously demonstrated a requirement for β-arrestin2 in the MOP-mediated activation of c-Src in DRG neurons^18^ in which DAMGO causes voltage-independent inhibition of N-type (Ca_V_2.2) VACCs *via* c-Src phosphorylation of C-terminal tyrosine 1747^14^. N-type channels are likely mediators of opioid inhibition of sIPSCs in the VTA, since their selective blockade increases dopamine levels in the accumbens through disinhibition of dopaminergic neurons^11^. Further work will be required to determine whether N-type VACCs in VTA neurons are targets of c-Src mediated inhibition by opioids.

Several behavioural effects of opioids are strongly influenced by β-arrestin2. Compared to WT mice, β-arr2−/− mice exhibit enhanced MOP-mediated analgesia, reduced morphine analgesic tolerance and increased sensitivity to the reinforcing effect of morphine^19, 40^. These behavioural changes could be the consequences of a loss of MOP endocytosis and/or desensitisation in β-arr2−/− mice. Indeed, either a lack of β-arrestin2 or the inhibition of c-Src reduces MOP endocytosis and desensitisation, respectively^18, 41^. However, some morphine stimulated behaviours are attenuated in β-arr2−/− mice, which cannot easily be explained by a deficiency in desensitisation and/or endocytosis. These include reductions in psychomotor stimulation, respiratory depression and constipation^35, 42^. Such behavioural deficits presumably reflect the need for β-arrestin2 in a signalling capacity^10^. β-Arrestins participate in several GPCR-mediated behaviors by recruiting various kinases and phosphatases. For example, morphine-evoked locomotor stimulation involves β-arrestin2-dependent recruitment of pERK to D1 receptors^35^. This psychomotor effect of morphine is attenuated in mice lacking either D1 receptors or β-arrestin2 and in WT mice treated systemically with the MEK inhibitor, SL327. In the current study we examined whether pERK participates in morphine’s inhibition of sIPSC frequency in VTA neurons. However, unlike PP2, which attenuated morphine’s inhibition of sIPSCs, SL327 did nothing implying that c-Src, rather than ERK, is involved in this β-arrestin2-mediated effect in VTA neurons.

Simplistically, our finding that a reduction in β-arrestin2/c-Src activity reduces morphine-evoked disinhibition of VTA neurons is at odds with the behavioural data that demonstrate enhanced sensitivity to the reinforcing effect of morphine in β-arr2−/− mice^40^. However, the phenotypic consequences of systemic abolition of β-arrestin2 are, of course, complicated by the multifaceted role of the protein in GPCR trafficking and function. The role of β-arrestin2 to recruit and signal through c-Src is only a single component of a complex array of activities^10^. In future, conditional deletion of β-arrestin2 in VTA inhibitory interneurons and focal delivery of c-Src inhibitors will help to establish the behavioral role of this pathway. It will also be important to establish whether reduced attenuation of IPSCs seen either in the absence of β-arrestin2, or in the presence of c-Src inhibition leads to reduced opioid-induced dopamine release in the striatum. This could be achieved in the slice by detecting dopamine electrochemically in the vicinity of the accumbal synapse.

Our demonstration that DOPs participate in morphine- and DPDPE-mediated inhibition of sIPSC frequency in VTA neurons is also important. DOP-mediated disinhibition of dopaminergic neurons presumably accounts for the previously reported intra-VTA self administration of DPDPE by rats^25^. The presence of MOPs and DOPs also raises the possibility of heteromers in VTA neurons^43^. MOP/DOP heteromers exhibit β-arrestin2 recruitment, desensitisation and signalling that differ from those of MOPs and DOPs expressed independently. We also demonstrated that the simultaneous recombinant expression of DOPs causes constitutive recruitment of β-arrestin2 to MOPs. It is therefore possible that in DOP−/− VTA neurons, MOP signalling is disrupted by reduced β-arrestin2 recruitment. However, there was no change in the inhibition of sIPSCs by DAMGO in DOP−/− neurons suggesting that MOP/DOP heteromers are not involved. DAMGO and morphine are both able to activate recombinant MOP/DOP heteromers^43^. By contrast to the VTA, there is evidence for oligomerization of MOPs and DOPs in DRG neurons in which an absence of DOPs reduces the efficacy of both DAMGO and morphine for inhibition of VACCs^44^. The most likely scenario in the VTA is an involvement of β-arrestin2 mediated inhibition of sIPSC frequency upon activation of MOPs by either morphine, or DAMGO, and DOPs by morphine. This would account for the reduction in the inhibitory effect of both opioids in β-arr2−/− VTA neurons. However, the evidence here for a lack of MOP/DOP heteromers is by no means definitive. Additional approaches to explore the possible involvement of heteromers in the VTA and other nuclei would be worthwhile. Future studies of slices from red-MOP/green-DOP double knock-in mice might provide an opportunity to directly visualise interactions between the two receptors^24^. Such an approach will be particularly helpful to establish the applicability of prior recombinant studies of opioid receptor oligomerisation to native receptors expressed by neurons.

It is well established that some ligands recruit distinct signalling pathways to GPCRs^45^. At the simplest level, differing selectivities of agonists for different opioid receptors (*e.g*. MOPs and DOPs in VTA neurons) could give rise to agonist specific modulation of the inhibitory control of dopaminergic neurons. Different ligands may also activate the same receptor recruiting alternative arrestins and this may correspond to their propensity to cause endocytosis. For example, the low-MOP internalising agonist morphine primarily recruits β-arrestin2, whereas the highly internalizing agonist DAMGO also recruits β-arrestin1^46^. This type of ligand-directed signalling may only become apparent during prolonged agonist exposure. The evidence from DRG neurons suggests that, like β-arrestin2, c-Src has both an immediate role in opioid receptor signalling and participates in longer term changes in receptor localisation^14, 18^.

The duration of exposure of neurons to opioids and other drugs in this *in vitro* study does not necessarily emulate that likely to occur *in vivo*. Each drug was typically applied to slices for 450 s with a maximum exposure time of 53 min when morphine was applied during concentration-response experiments. *In vivo* exposures of opioids would be longer, closer to those used in the β-arrestin2 recruitment aspect of our study. In future studies it would be informative to examine the time course of opioid effects on VTA neurons to examine desensitization and tolerance.

In this study we established a role for β-arrestin2/c-Src signalling in the inhibition of sIPSCs following activation of MOPs. Our data suggest that β-arrestin2-mediated activation of c-Src in the VTA contributes to disinhibition of dopaminergic neurons by opioids. There are numerous targets of c-Src mediated phosphorylation that may be involved in the short and longer term effects of opioids including N-type VACCs and NMDA receptors, respectively^14, 47^. Furthermore, c-Jun N-terminal kinase is another target of c-Src phosphorylation implicated in several behavioural effects of morphine, including tolerance^17, 48, 49^. Therefore, it is possible that in addition to its acute role in opioid inhibition of IPSC frequency, identified here, through recruitment of additional targets, c-Src may contribute to longer term changes in neuronal function.

## Methods

For additional details, please see Supplementary Materials and Methods. Unless otherwise stated, reagents were from Sigma-Aldrich (Dorset, UK).

### Animals

All methods were carried out in accordance with UK Home Office guidelines and regulations using approved procedures. All experimental protocols were approved by both the University of Dundee ethical review committee and the Home Office. Mouse lines were fully backcrossed onto the C57BL/6J background from Jackson Labs. MOP−/− mice were originally generated by Matthes *et al*.^2^ and are available from Jackson Labs (stock number 007559). DOP−/− mice were generated by Filliol *et al*., 2000 (stock number 007557). The βarr2−/− mice used were developed by Bohn *et al*.^19^ (stock number 011130). βarr2−/−//DOP−/− mice were generated by crossing βarr2−/− and DOP−/− mice and were maintained on the C57BL/6J background. Wild type (WT) C57BL/6J mice (Charles River, Kent, UK) were periodically bred with knockout animals and were genotyped by Transnetyx (Cordova, TN, USA). Mice were maintained, bred and sacrificed in accordance with UK Home Office regulations.

### Mouse genotyping

Genotyping was performed on genomic DNA extracted from ear clippings of mice older than six weeks of age. For mice sacrificed for electrophysiology experiments, ear clippings were collected post mortem. These were sent to Transnetyx (Cordova, TN, USA) for genotyping. In many cases genotypes of breeding pairs were established providing knowledge of the genotypes of offspring used in subsequent experiments; therefore the investigator was not systematically blinded to mouse genotype.

### Brain Slice Electrophysiology

Mouse brain slices were prepared from C57BL/6 J mice aged postnatal day 17–21 killed by cervical dislocation. Brains were removed and placed in ice cold slicing solution bubbled with 95% O_2_/5% CO_2_. The slicing solution comprised: 234 mM sucrose, 26 mM NaHCO_3_, 10 mM glucose, 10 mM MgSO_4_, 2.5 mM KCl, 1.3 mM NaH_2_PO_4_, and 0.5 mM CaCl_2_. Horizontal slices (250 μm) containing the VTA were cut in ice cold sucrose solution using a Leica VT100S vibratome (Milton Keynes, UK). Slices corresponding to bregma −4.12 to bregma −4.44^50^ were incubated in extracellular solution (126 mM NaCl, 26 mM NaHCO_3_, 10 mM Glucose, 3 mM KCl, 2 mM MgCl_2_, 2 mM CaCl_2_, 1.3 mM NaH_2_PO_4_), bubbled with 95% O_2_/5% CO_2_ for ≥1 hr at room temperature. Kynurenic acid (2 mM; Abcam, Cambridge, UK) was added to inhibit ionotropic glutamatergic events. In recordings of miniature IPSCs (mIPSCs), tetrodotoxin (TTX; 500 nM) was added. Borosilicate glass recording pipettes were filled with intracellular solution (138 mM CsCl, 10 mM HEPES, 10 mM EGTA, 1 mM CaCl_2_, 2 mM MgCl_2_, 2 mM Mg-ATP, 5 mM QX-314 (Tocris, Bristol, UK) - pH 7.4 with CsOH) and had resistances between 2–5 MΩ. Recordings were made from slices superfused with oxygenated (95% O_2_/5% CO_2_) extracellular solution maintained at 36 °C (HPT-2 heated perfusion tube, ALA Scientific, Farmingdale, NY, USA). Whole-cell voltage-clamp recordings, filtered at 5 kHz and digitised at 10 kHz (NI USB-6221 interface, National Instruments, Newbury, UK), were obtained at a holding potential of −60 mV (Axopatch 200B, Molecular Devices, Sunnyvale, CA, USA) and acquired using Strathclyde Electrophysiology Software (John Dempster, University of Strathclyde).

### Immunohistochemistry

In some cases individual neurons in electrophysiological experiments were labelled with biocytin prior to slice fixation. Biocytin (1%) was included in the intracellular solution in the recording pipette and allowed to diffuse into the voltage-clamped neuron enabling its subsequent identification. Brain slices were fixed overnight in a 1% paraformaldehyde (PFA) solution containing picric acid (TAAB Labs, Berks, UK). After fixation slices were washed three times in 0.1 M phosphate buffer containing 0.05% sodium azide. Cells in brain slices were permeabilised in 0.1 M phosphate buffer containing 0.3% Triton × 100 (PBT). Slices were blocked in PBT supplemented with 10% bovine serum. Rabbit anti-tyrosine hydroxylase primary antibody (AB152, Merck Millipore, Watford, UK) was used to label DA neurons at a dilution of 1:1000. Primary antibody labelled TH was detected using anti-rabbit IgG conjugated Alexa Fluor 594 (A-21207, Invitrogen, Paisley, UK) at a dilution of 1:1000. Biocytin was detected using streptavidin conjugated Alexa Fluor 488 (S-11223, Invitrogen) at a dilution of 1:200. Labelled slices were mounted on slides with FluorSave reagent (Merck Millipore). Slides were imaged using confocal microscopy (Leica TCS SP-5 confocal microscope). The TH signal (AlexaFluor 594) was excited at 594 nm and the emitted fluorescence was collected between 600 nm and 650 nm. The biocytin signal (streptavidin conjugated AlexaFluor 488) was excited at 488 nm and the emitted fluorescence was collected between 500 nm and 550 nm. Images were obtained at 10x and 40x magnification.

### Drug application

Spontaneous inhibitory postsynaptic currents (sIPSC) were recorded in the absence and presence of a variety of drugs including bicuculline, morphine, DAMGO, DPDPE (Abcam, Cambridge, UK), PP2, PP3 and SL327 (all from Tocris, Bristol, UK). All drug solutions were prepared as required on the day of use from frozen stock solutions and were diluted in the extracellular solution. Control and drug containing solutions were applied to the recording chamber using a peristaltic pump (Scientifica, East Sussex, UK). DAMGO and morphine were applied at ascending concentrations to establish the concentration-response relationships. Each concentration was applied to slices for 450 s to establish steady state; with up to seven morphine concentrations per neuron, there was a maximum of 53 min of exposure.

### Analysis

Electrophysiological data were analysed offline using Strathclyde Electrophysiology Software, WinEDR and WinWCP (courtesy of Dr John Dempster, University of Strathclyde, Glasgow, UK). Each recording segment (*i.e*. control, or drug) consisted of at least 450 s, this was then divided into three 120 s segments and the event frequency of each segment was determined from the inter-event interval and averaged for each cell. Frequencies were averaged across several cells (as indicated by n values provided in the text). There was an absence of apparent burst activity as evidenced by stable event frequencies. IPSCs were threshold detected (amplitude −4 pA, duration 3 ms) and visually inspected, a minimum of 50 events *per* recorded segment were used for kinetic analysis. Peak amplitudes, rise times and decay times were determined for each IPSC, averaged for each cell and then averaged across several cells (as indicated by n values in the text). The decay phase of the averaged IPSCs (from each control/drug treated segment) were best fitted (98–5% of the peak amplitude) with a double exponential function (Equation 1):

1 $$f(t)=Af\,\times \,{e}^{\frac{-t}{\tau f}}+As\times {e}^{\frac{-t}{\tau s}}$$

where t was time, Af and As were the amplitudes of the fast and slow time constants (τf and τs), respectively. A weighted decay time constant (τw) was also calculated for the averaged events according to (Equation 2):

2 $$\tau w=\tau f\times Pf+\tau s\times Ps$$

where τf and τs are the fast and slow decay time constants and Pf and Ps are the proportions of the decay relative to each of the component parts (Maguire *et al*., 2014).

### Statistics

Slices from at least three animals were used to obtain electrophysiological data under each condition. The n numbers represent the number of distinct voltage-clamped VTA neurons from which currents were recorded. Data are expressed as mean ± standard error of mean (SEM) unless otherwise stated. Statistical comparisons were made using the Student t-test or ANOVA with *post hoc* Tukey’s or Dunnett’s tests as stated. EC_50_ values were determined from fitting the logistic equation to concentration-response relationships (GraphPad Prism, La Jolla, CA, USA). From these fits 95% confidence intervals (CIs) were determined. EC_50_ values were considered significantly different in cases where there was no overlap between CIs.

## Electronic supplementary material

Supplementary Material
