# Supplementary material for: Morphine activation of mu opioid receptors causes disinhibition of neurons in the ventral tegmental area mediated by β-arrestin2 and c-Src
Source: Sci Rep. 2017 Aug 30;7:9969. doi: 10.1038/s41598-017-10360-8 (PMC5577270; doi:10.1038/s41598-017-10360-8)
Supplement: Supplementary file 1 — Supplementary Material [file 41598_2017_10360_MOESM1_ESM.pdf]

Morphine activation of mu opioid receptors causes disinhibition of neurons in the ventral tegmental area mediated by β-arrestin2 and c-Src

Fiona A. Bull, Daniel T. Baptista-Hon, Jeremy J. Lambert, Wendy Walwyn & Tim G. Hales

Supplementary Materials and Methods

Cell culture and transfection

PathHunter CHO cells (DiscoverX) stably expressing β-galactosidase complementation fragments tagged MOPs and β-arr2 were routinely maintained as per manufacturer's instructions. Transfections were performed on cells plated on 35 mm dishes with lipofectamine and 2 µg of either empty vector (pCDNA3.1) or human DOP cDNA. Transfection efficiency was determined with parallel transfections using 2 µg eGFP cDNA.

PathHunter protein fragment complementation assay

Mock or DOP transfected PathHunter CHO cells were seeded onto 96 half well plates at a density of 5000 cells well^{-1} and left to settle overnight. DAMGO and morphine were added to the wells using serial dilution. The final concentration of DAMGO and morphine ranged between 0.3 nM to 30 µM. Cells were incubated with agonists for 90 mins to effect changes in β-arrestin2 recruitment. Agonist free luminescence was measured from wells not challenged with agonists. PathHunter Detection kit reagents (DiscoverX), which contain the substrate for β-galactosidase were then added and incubated for 60 mins. Chemiluminescence as a result of enzymatic activity was detected using Flexstation 3 plate reader at 1 s integration.

Data analysis

Chemiluminescence from PathHunter assays were recorded as relative luminescence units (RLU), and concentration-response data were normalised to maximum. A logistics function was fitted to concentration response data to obtain agonist EC_{50} and Hill slope (n^H) values. The logistics function is defined by (Equation 3):

$$f([Agonist]) = \frac{100}{1 + 10^{(logEC_{50} - [Agonist]) \times n^H)}}$$

All data are presented as mean ± SEM of n number of plates. Statistical analyses were performed using the t-test.

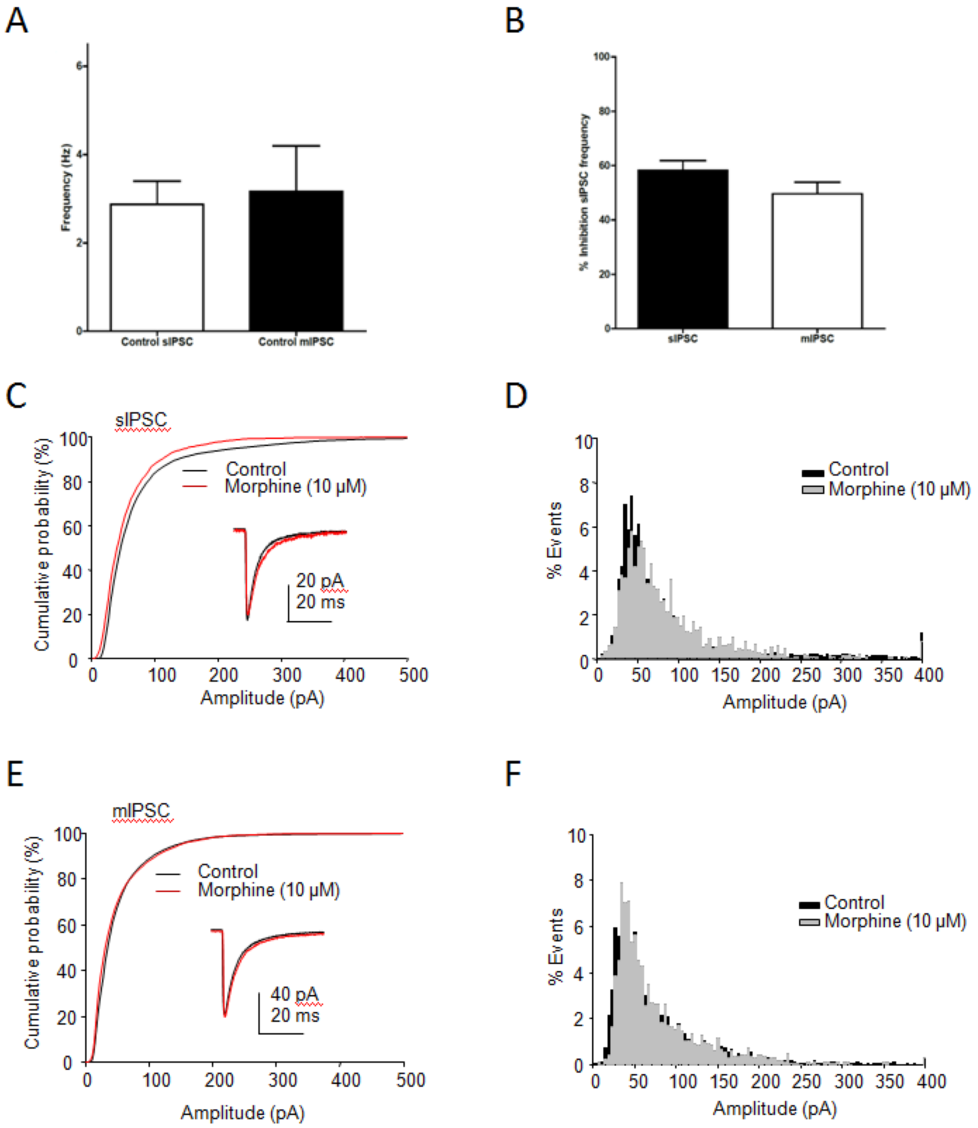

Supplementary Figure 1. Morphine did not affect the amplitudes of IPSCs either in the absence or presence of TTX. A, The frequency of sIPSCs was unaffected by the application of TTX (500 nM) applied to the recording chamber. B, TTX also had no significant effect on the inhibition of sIPSC frequency by morphine (10 µM). Morphine was applied in the absence (sIPSC) and presence (mIPSC) of TTX (500 nM). C, The exemplar cumulative probability, with averaged sIPSCs (red – in the presence of morphine) and D, amplitude histogram plots illustrate the lack of effect of morphine on sIPSC amplitude. Likewise E, cumulative probability, with averaged mIPSCs (red – in the presence of morphine) and F, amplitude histogram plots reveal a lack of effect of morphine on the amplitudes of mIPSCs. Average IPSC amplitude and kinetic data are provided in Supplementary Table 1.

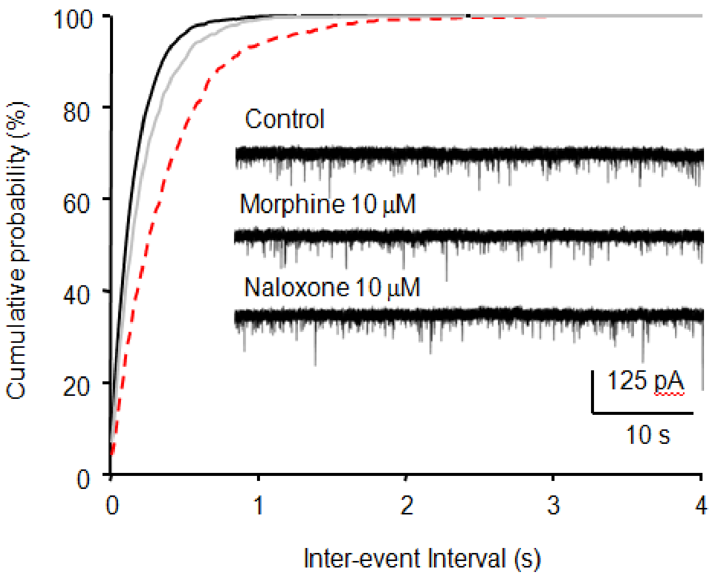

Supplementary Figure 2. Naloxone partially reverses the decrease of sIPSC frequency caused by morphine. Exemplar cumulative probability curves represent inter-event interval data from sIPSCs recorded under control (black), morphine (red) and naloxone washout conditions from the same neuron.

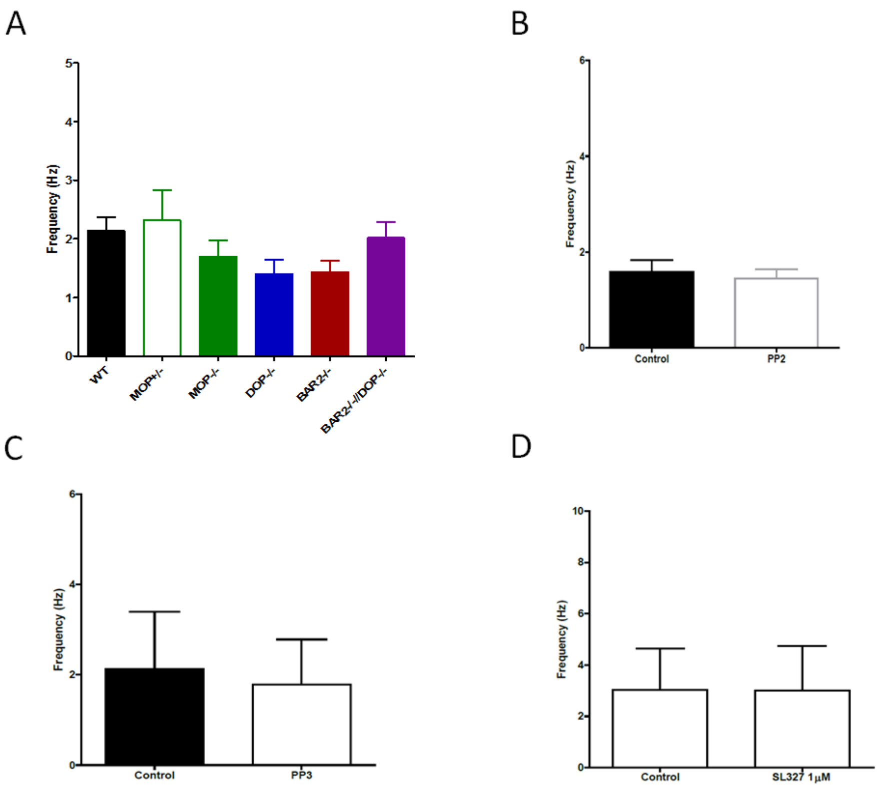

Supplementary Figure 3. Neither genotype nor kinase inhibitor application to WT VTA neurons affected basal sIPSC frequency. A, There was no significant difference in the basal sIPSC frequencies recorded from any of the mouse strains used in this study. B - D, The application of PP2 (10 µM), PP3 (10 µM) or SL327 (1 µM), respectively, had no effect on sIPSC frequency. Statistical comparisons were by one way ANOVA (A) and the paired Student's t-test (B - D).

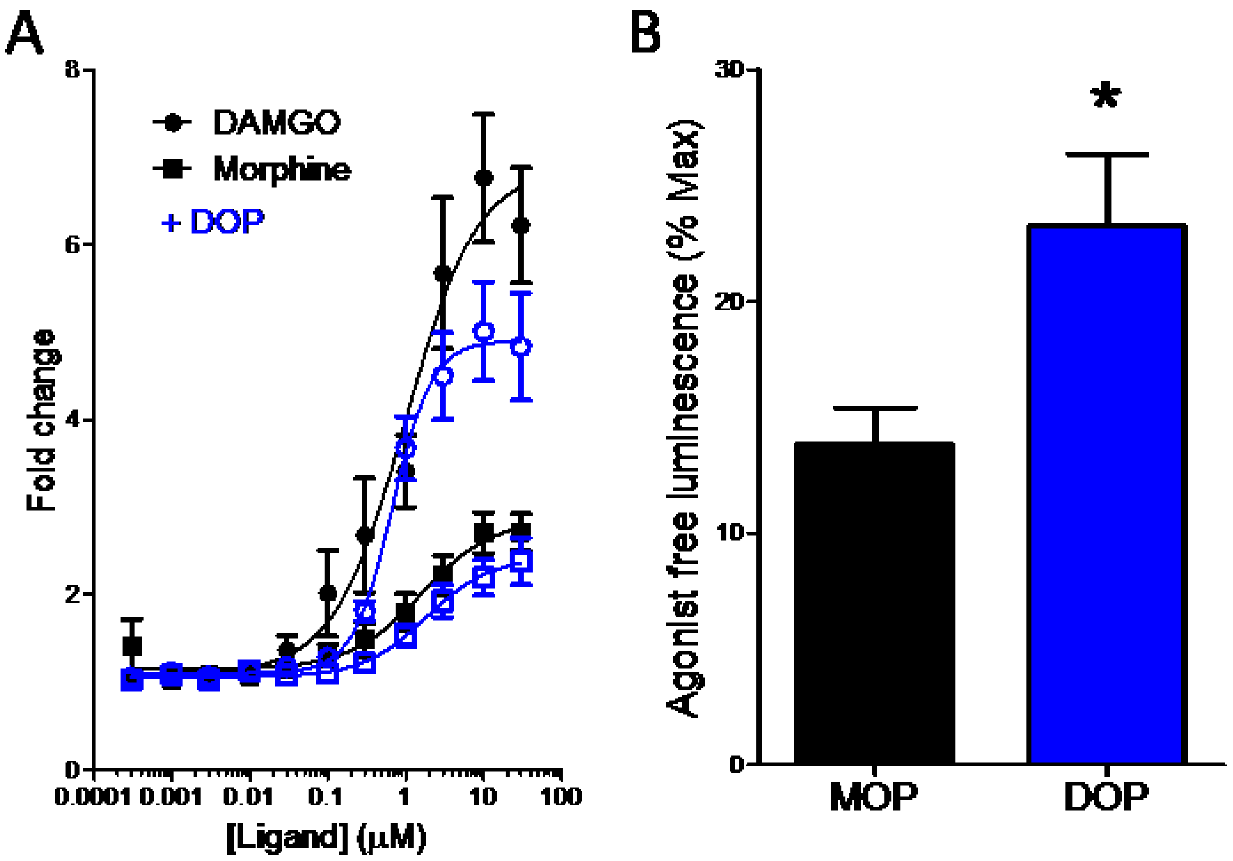

Supplementary Figure 4. DOPs influence β-arrestin2 recruitment to MOPs. A. Concentration-response relationships for β-arrestin2 recruitment in the presence (blue; n = 20) or absence (black; n = 20) of DOPs. The fitted curve represents the logistics function fit to the data. Individual logistics function fits to concentration-response data yielded DAMGO EC_{50} values of 1.5 ± 0.20 μM (n = 20) in the absence of DOPs and 0.85 ± 0.13 μM (n = 20) in the presence of DOPs. The presence of DOP significantly increased the potency of DAMGO (P = 0.01; t-test). The EC_{50} of morphine was 3.2 ± 0.82 μM (n = 20) in the absence of DOPs and 3.9 ± 1.7 μM (n = 20) in the presence of DOPs. The presence of DOP did not reduce the potency of morphine (P = 0.73; t-test). DAMGO hill slope values were 1.8 ± 0.38 and 2.3 ± 0.26 in the absence and presence of DOPs. Morphine Hill slope values were 1.6 ± 0.23 and 1.8 ± 0.32 in the absence and presence of DOPs. (B) The presence of DOPs significantly increased agonist free luminescence values (expressed as % maximum DAMGO-evoked luminescence) from 14 ± 1.6 % (n = 20) in the absence of DOPs to 23 ± 3.1 % (n = 20) in the presence of DOPs (*P = 0.01; t-test). Consistent with the prior work of Rozenfeld and Devi (2007), these data suggest that expression of DOPs causes an increase in the agonist independent recruitment of β-arrestin2 to MOPs.

References

Maguire EP, Macpherson T, Swinny JD, Dixon CI, Herd MB, Belelli D, Stephens DN, King SL, Lambert JJ (2014). Tonic inhibition of accumbal spiny neurons by extrasynaptic alpha4betadelta GABAA receptors modulates the actions of psychostimulants. *J Neurosci* **34**: 823-838.

Rozenfeld R, Devi LA (2007). Receptor heterodimerization leads to a switch in signaling: β-arrestin2-mediated ERK activation by mu-delta opioid receptor heterodimers. *FASEB J* **21**: 2455-2465.

	sIPSC		mIPSC	
	Control	Morphine 10 µM	Control	Morphine 10 µM
Peak Amplitude (pA)	-83.7 ± 6.8	-94.8 ± 13.6	-81.1 ± 8.9	-86.6 ± 9.3
Rise time (ms)	0.64 ± 0.02	0.65 ± 0.04	0.64 ± 0.03	0.67 ± 0.03
T70 (ms)	7.5 ± 0.4	7.6 ± 0.6	7.4 ± 0.7	8.1 ± 0.8
Tau ω (ms)	6.5 ± 0.4	6.8 ± 0.7	7.0 ± 1.0	6.8 ± 0.7

Supplementary Table 1. Parameters of miniature and spontaneous IPSCs recorded from VTA neurons. Morphine inhibits IPSC frequency (see Table 1), but does not alter the kinetics of the events. There are no significant changes in the kinetic parameters or amplitudes of the IPSC events in the presence of TTX (500 nM) or morphine (10 µM). For the sIPSC recordings n = 8 and for the mIPSC recordings n = 12 (see Analysis in Methods).
